# Live poultry market closure and avian influenza A (H7N9) infection in cities of China, 2013–2017: an ecological study

**DOI:** 10.1186/s12879-020-05091-7

**Published:** 2020-05-24

**Authors:** Ying Chen, Jian Cheng, Zhiwei Xu, Wenbiao Hu, Jiahai Lu

**Affiliations:** 1 School of Public Health, Key Laboratory of Tropical Diseases Control of Ministry of Education, One Health Center of Excellence for Research &Training, Sun Yat-sen University, Guangzhou, China; 2 School of Public Health and Social Work, Institute of Health and Biomedical Innovation, Queensland University of Technology, Brisbane, Australia

**Keywords:** Avian influenza A (H7N9), Live poultry market closure, Effect evaluation, CARTs

## Abstract

**Background:**

Previous studies have proven that the closure of live poultry markets (LPMs) was an effective intervention to reduce human risk of avian influenza A (H7N9) infection, but evidence is limited on the impact of scale and duration of LPMs closure on the transmission of H7N9.

**Method:**

Five cities (i.e., Shanghai, Suzhou, Shenzhen, Guangzhou and Hangzhou) with the largest number of H7N9 cases in mainland China from 2013 to 2017 were selected in this study. Data on laboratory-confirmed H7N9 human cases in those five cities were obtained from the Chinese National Influenza Centre. The detailed information of LPMs closure (i.e., area and duration) was obtained from the Ministry of Agriculture. We used a generalized linear model with a Poisson link to estimate the effect of LPMs closure, reported as relative risk reduction (RRR). We used classification and regression trees (CARTs) model to select and quantify the dominant factor of H7N9 infection.

**Results:**

All five cities implemented the LPMs closure, and the risk of H7N9 infection decreased significantly after LPMs closure with RRR ranging from 0.80 to 0.93. Respectively, a long-term LPMs closure for 10–13 weeks elicited a sustained and highly significant risk reduction of H7N9 infection (RRR = 0.98). Short-time LPMs closure with 2 weeks in every epidemic did not reduce the risk of H7N9 infection (*p* > 0.05). Partially closed LPMs in some suburbs contributed only 35% for reduction rate (RRR = 0.35). Shenzhen implemented partial closure for first 3 epidemics (*p* > 0.05) and all closure in the latest 2 epidemic waves (RRR = 0.64).

**Conclusion:**

Our findings suggest that LPMs all closure in whole city can be a highly effective measure comparing with partial closure (i.e. only urban closure, suburb and rural remain open). Extend the duration of closure and consider permanently closing the LPMs will help improve the control effect. The effect of LPMs closure seems greater than that of meteorology on H7N9 transmission.

## Background

Avian Influenza is a zoonosis spread from live poultry directly. Since the first laboratory-confirmed human infection with avian influenza A (H7N9) was reported on 31 March 2013, epidemics of human infections of H7N9 virus in mainland China have generally followed a seasonal trend, with five previous major outbreaks on record [1]. In the epidemic of the past 5 years, human cases of H7N9 have an annual peak in January and February, and the mortality rate was around 40% [2]. Among them, the epidemic situation was the worst in the 5th wave in 2017, and 766 laboratory-confirmed cases were observed (nearly half of the total number of H7N9 cases reported in 2013–2017 [3]). The seasonality of H7N9 epidemics implies that ambient temperature and absolute humidity had significant independent and interactive effects on H7N9 infection risks [4].

Besides, exposure to live poultry is another main risk factor for H7N9 infection. One of the main ways people are exposed to the H7N9 virus is through exposure to live poultry markets (LPMs). Previous studies found that 80% of households reported purchasing poultry at LPMs at least once a year in 2006 [5], and 47% of residents in Guangzhou city reported visiting an LPMs > = 1 times a year in 2013. Retail and wholesale markets are two major types of LPMs in China. A prior study suggested that the retail but not the wholesale LPMs are strongly associated with human H7N9 infection risk [6, 7]. Given that there has been no evidence of H7N9 virus spread through human-to-human so far, the preventive measure should still focus on the human to animal [8]. In rural areas, 48% of respondents reported that they raised backyard poultry [9], while for cities the highest influenza A positive rates were detected at the retail LPMs [6].

To prevent the poultry-to-human transmission of this novel virus, many cities in the pearl and Yangtze river deltas implemented the LPMs closure. Once the confirmed H7N9 cases were reported in a city, the government implemented the LPMs closure and slaughtered or transferred the live poultries to decrease the disease-causing contact chance, and the numbers of new-onset human H7N9 viral infections decreased rapidly [10–12]. For instance, one strict prevention and control measures were used to conduct in Guangdong Province, which was daily cleaning of the market, weekly disinfection of the market and monthly closings of the live market. Some cities adopted a permanent closure of LPMs in recent years, such as in Shenzhen, and other cities have been taking the LPMs closed for a certain period in each year, like Shanghai and Guangzhou city. While for Hangzhou and Suzhou city implemented the closure only in urban area of cities. In short, the LPMs measures of closure are different when implemented in each city.

Although the LPMs closures were highly effective in preventing H7N9 viral infections in humans by substantially reducing human exposure to poultry, which has been proved by many previous studies [12–15]. In fact, most of the previously closed LPMs have reopened after the outbreak, due to the enormous economic and political pressures otherwise [16]. Hence, there is an urgent need for dissemination of the optimal available scientific evidence on the effectiveness of LPMs closure to help policymakers reassess intervention strategy in future human outbreaks of influenza A (H7N9). The paper aims to quantify the effect of LPMs closure on H7N9 infection after adjustment of climate variables in various cities and the role of LPMs closure of different scale and duration, to provide more empirical evidences for policy-makers for the efficient control of H7N9.

## Method

### Data source

We collected the laboratory-confirmed human cases of avian influenza A (H7N9) virus infection during 2013–2017 from the Chinese National Influenza Centre (http://www.chinaivdc.cn/cnic/zyzx/lgzb/) and the Centre for Health Protection of Hong Kong (https://www.chp.gov.hk/en/index.html), which built an integrated dataset with detailed demographic, epidemiology, and geographical information for every case. We extracted information about the location and date of illness confirmation.

Considering the ecological study needs enough sample size, we selected the five cities with the most H7N9 cases in the country as the study sites for respective analysis. Information of LPMs intervention about closing measures start and end time, the area of closing implementation (urban or suburb area) were collected from the Ministry of Agriculture of China and Agricultural Bulletin in each city. We also checked this information with official media coverage of each city as verification and supplement. Daily meteorological data for five cities were obtained from the China Meteorological Data Service Center (http://data.cma.cn/en). The meteorological data included daily mean temperature (TEMP), relative humidity (RHU), which were used to explore the joint effects of weather factors on H7N9 transmission.

### Statistical analysis

The specific exposure date was not collected by the data source. We calculated the exposure date to H7N9 virus for each case based on the confirmed dates of cases we had. The days from exposure to confirmed days was defined as: days from infection to onset (3.5) + days from onset to hospital admission (3) + days from hospital admission to laboratory-confirmed (7.5) = about 14 days (2 weeks) [8, 17, 18]. The timelines for each city were derived by matching the calculated live poultry exposure dates of the cases with the start and end dates of the LPMs closure on the time axis. We correspond to the peak periods of H7N9 epidemic in different cities as the respective study periods, that is, from exposure date of the first case or the start date of the LPMs closure in each city, to exposure date the last case or the reopening date of the LPMs.

We used the generalized linear model with a Poisson link to derive city-specific estimates of LPMs closure. Based on the estimated risk coefficients (β) for each city, relative risks (RR) in each period i were computed through RR_i_ = exp. (β_i_) and relative risk reduction (RRR) = 1 − RR were calculated [19]. In this daily time series, independent variables are divided into three categories: 1 – before LPMs closure, 2 – during or after LPMs all closure (all LPMs be closed in whole city), and 3 – during or after partial LPMs closure (only LPMs in urban be closed, suburban and rural area remain open); and the dependent variable was the number of cases on each day. Considering that LPMs in Suzhou and Hangzhou were partial closed i.e. only in urban area, we calculated the RR by merging the data according to the implemented or non-implemented area and date, to evaluate the effect of closure scale. Moreover, we controlled for the confounding effects of the current day’s relative humidity, and the mean temperature. Hence, the GLM model is given as follows:

$$ {\mathsf{Y}}_{\mathsf{t}}\sim \mathsf{Poisson}\ \left({\mathsf{\mu}}_{\mathsf{t}}\right) $$

$$ \mathsf{Log}\ \left({\mathsf{\mu}}_{\mathsf{t}}\right)=\mathsf{\beta 0}+\mathsf{\beta}\mathsf{1}\left(\mathsf{Intervention}\right)+\mathsf{\beta}\mathsf{2}\mathsf{MTem}+\mathsf{\beta}\mathsf{3}\mathsf{RHum} $$

Where *t* is the day of the observatio*n; Y*_*t*_ is the observed daily cases number of H7N9 on day *t;* “*β0*” is the intercept; “Intervention” is the independent variables with three categories as mentioned above; “MTem” is the daily mean temperature; “RHum” is the daily mean relative humidity. Following this method, we quantitatively estimated the effect of LPMs closure implemented for cities.

In this study, we used regression tree to determine dominant factors and the hierarchical relationship of LPMs closure implemented and meteorological factors and their thresholds for H7N9 transmission. Classification and regression trees (CARTs) are a non-parametric statistical method, which is a kind of machine learning method. The dependent variable can be a categorical (classification tree) or a continuous variable (regression tree [20]. In our analysis, the root of tree is daily cases number and to split the tree to produce two and more child nodes, aiming to find out the most dominant factors through maximize the homogeneity among outcomes classified in each child node and maximize the heterogeneity between these two nodes. There are three factors in this regression tree: LPMs closure, daily mean temperature (TEMP), relative humidity (RHU). The splitter with variable and threshold near the root indicates it has higher decisive for the outcomes [21].

The statistical analysis was conducted with R statistical software (Version 3.5.2.), using packages mgcv, rpart and ggplot2. The graphical maps were performed by ArcMap 10.6 (version 10.6, ESRI Inc.). Statistical significance was set at *P* < 0.05 (two-tailed test).

## Results

### The overview of the study sites on LPMs closure of five cities

**Fig. 1:**
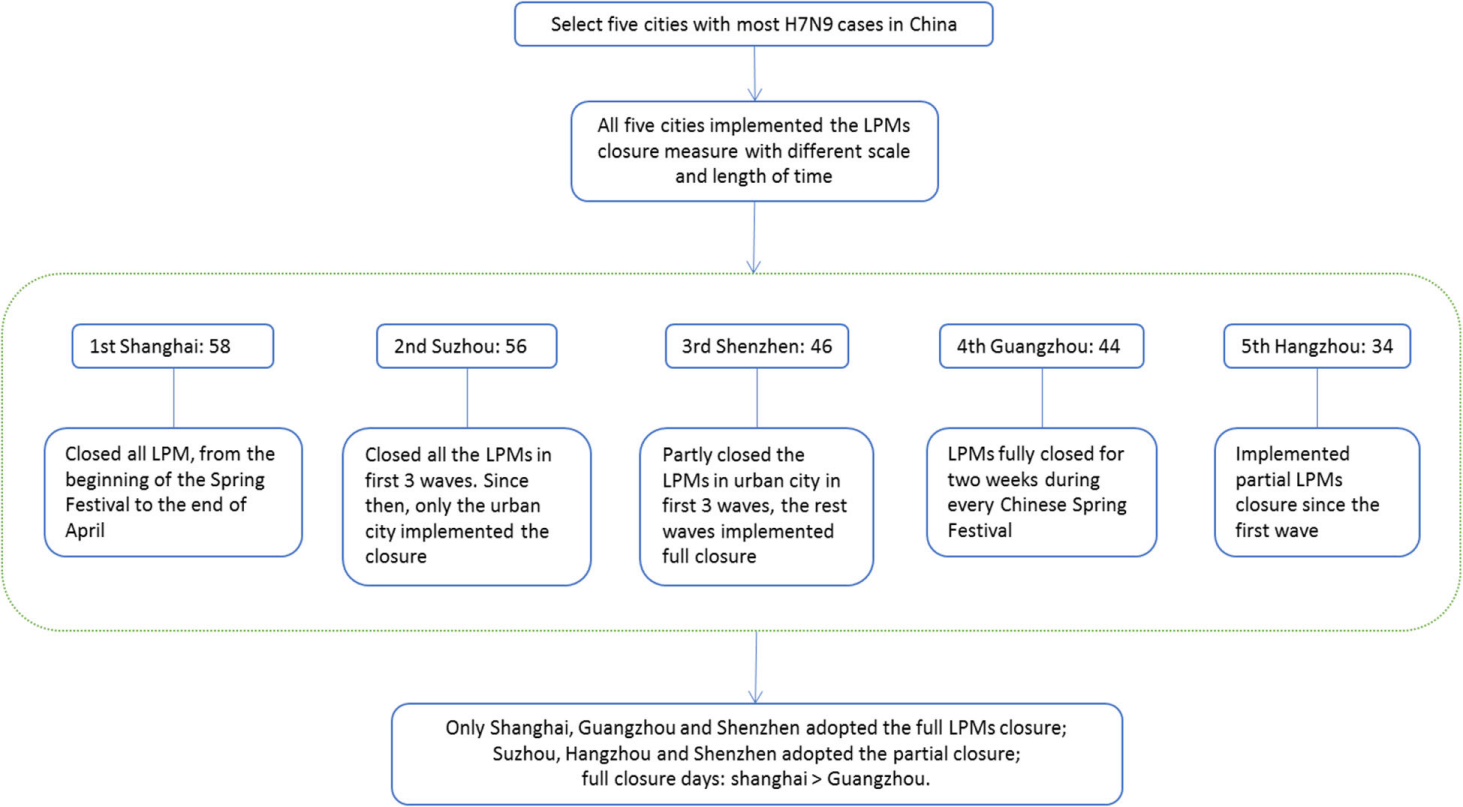
The overview of the study site for access the effect on LPMs closure for control H7N9 infection. The five cities’ name, number of H7N9 cases and LPMs (live poultry markets) closure time are show in boxes

A total of 1560 H7N9 cases were reported in China from 2013 to 2017 including 5 epidemic waves with 88% (30/34) provinces involved. The first epidemic wave was from Jan 1st to Sept 30th, 2013, and the subsequent three waves were from Oct 1st of 2013 to Sept 30th of 2014, Oct 1st of 2014 to Sept 30th of 2015, and Oct 1st of 2015 to Sept 30th of 2016. The last wave was from Oct 1st, 2016 to Dec 31st, 2017 [1, 22]. The five top cities with the most H7N9 cases were Shanghai, Suzhou, Shenzhen, Guangzhou, and Hangzhou. They located in Yangtze River Delta and Pearl River Delta, which are well-developed cities with large population density (Fig. 1).

**Fig. 2:**
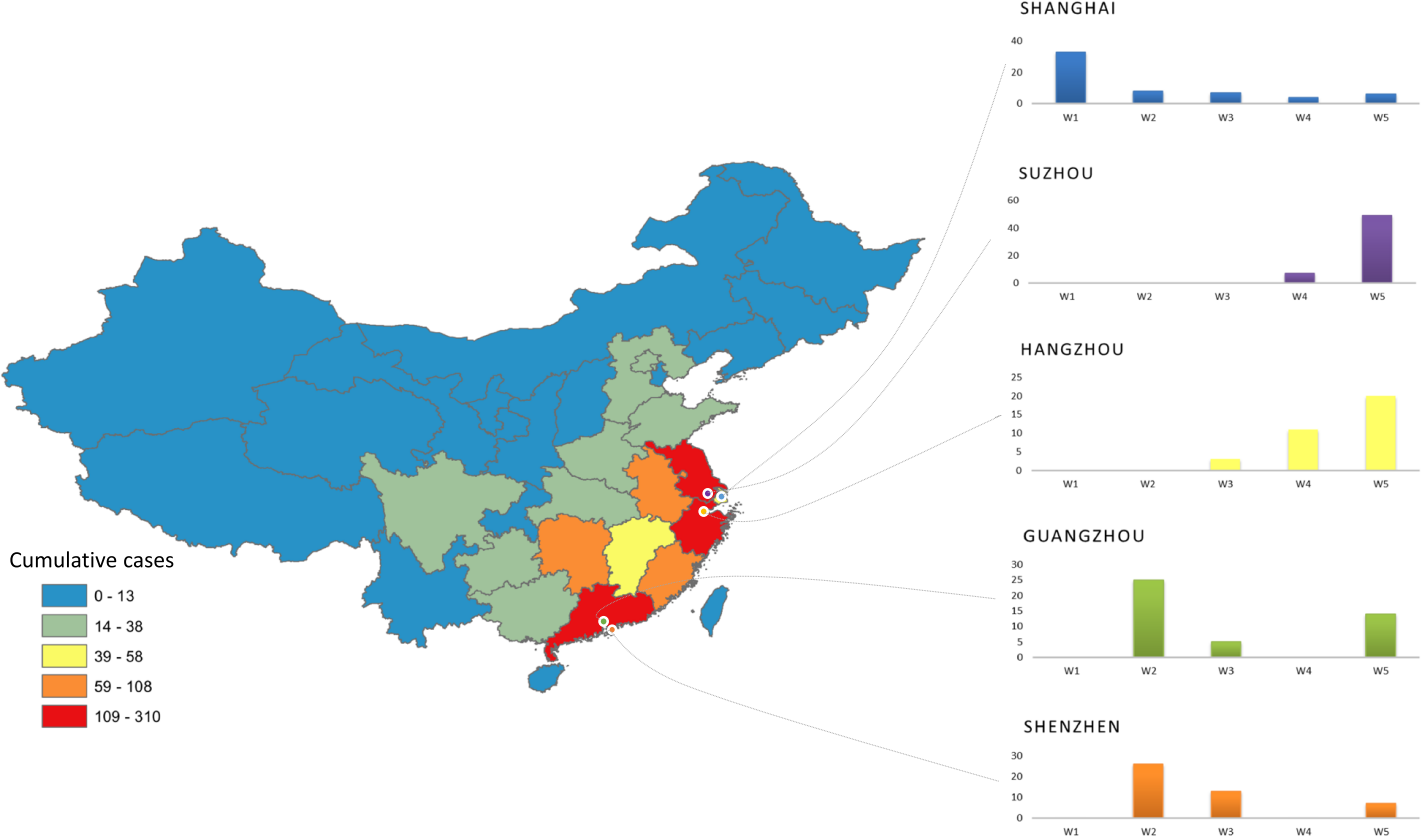
Geographical distribution of five cities with most H7N9 cases in China and their time trend of cases number from 1st wave to 5th wave (2013–2017)

**Fig. 3:**
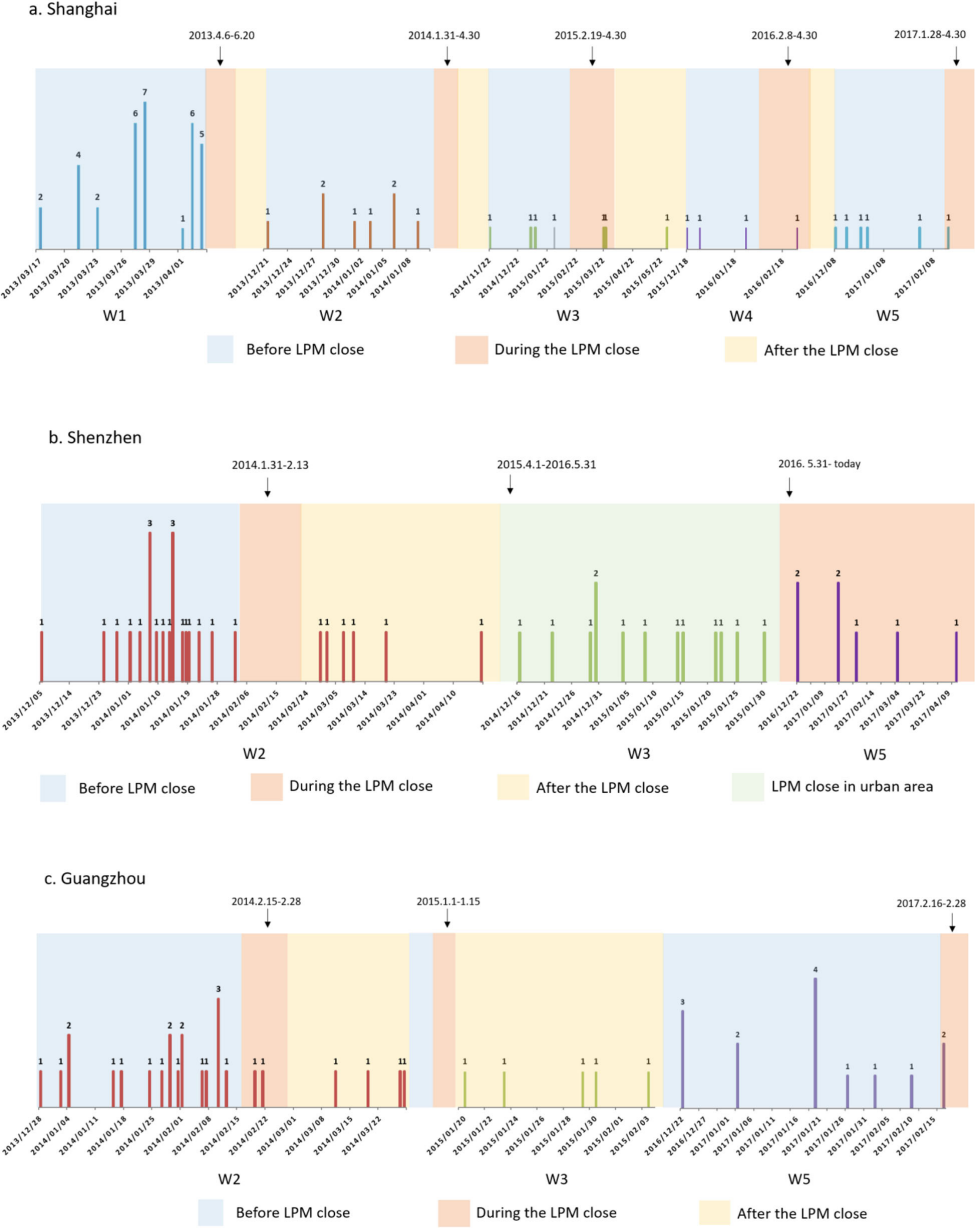
The timeline of LPMs closure in Shanghai (**a**), Shenzhen (**b**), and Guangzhou (**c**). Bars indicated the H7N9 case number, the various color in background indicated the different periods of LPMs closure. W1 refers to the wave of 1st epidemic, etc.

We can find that all five cities implemented the LPMs closure measure with different areas and length of time during 5 epidemic waves. Three cities are around the Yangtze River Delta: 1) In Shanghai, all LPMs were closed from the beginning of the Spring Festive to the end of April in each wave; 2) In Suzhou, closed all the LPMs in the first 3 waves, since then, the closure measure only was implemented in urban area of city; 3) In Hangzhou, LPMs closure implemented partially in urban area during five waves. The other two cities are located in Pearl River Delta: 1) Shenzhen, partially closed the LPMs in urban area during first 3 waves, the rest waves adopted the permanently all closure in the whole city till today; 2) Guangzhou, adopted partial LPMs closure in urban area during five waves. In summary, only Shanghai, Guangzhou and Shenzhen (latest 2 waves) implemented the LPMs all closure, Suzhou, Hangzhou and Shenzhen (first 3 waves) adopted the partial closure; comparing the all closure days in 5 waves we can find, Shanghai> Shenzhen> Guangzhou (Fig. 2). Timelines with exposure dates of each case, and days before during and after intervention in three cities (in different background color) were drawn respectively (Fig. 3), and the distribution of different periods lists in Table 1. Furthermore, general linear regression analysis shown a linear relationship in Shanghai between LPMs closure days (X) and epidemics interval days (Y) that is Y = 6.24X-224.37, *R*^*2*^ = 0.93, which indicated that longer the LPMs closure, later the next epidemic comes.

**Table 1:** Distribution of H7N9 cases at different periods of intervention in the three cities

	Shanghai (total = 58)					Shenzhen (total = 46)			Guangzhou (total = 44)		
	W1	W2	W3	W4	W5	W2	W3	W5	W2	W3	W5
Number of cases	33	8	7	4	6	26	13	7	25	5	14
Days of waves	18	27	188	72	72	135	46	114	91	15	58
Days between two waves	259	310	204	285	365	–	242	–	–	298	–
Number of cases before closure	33	8	4	3	4	19	–	–	19	0	12
Number of cases during closure	0	0	2	1	2	1	13	7	2	0	2
Number of cases after closure	0	0	1	0	0	6	–	0	4	5	0

### Relative risk reduction of H7N9 case infection through interventions

**Fig. 4:**
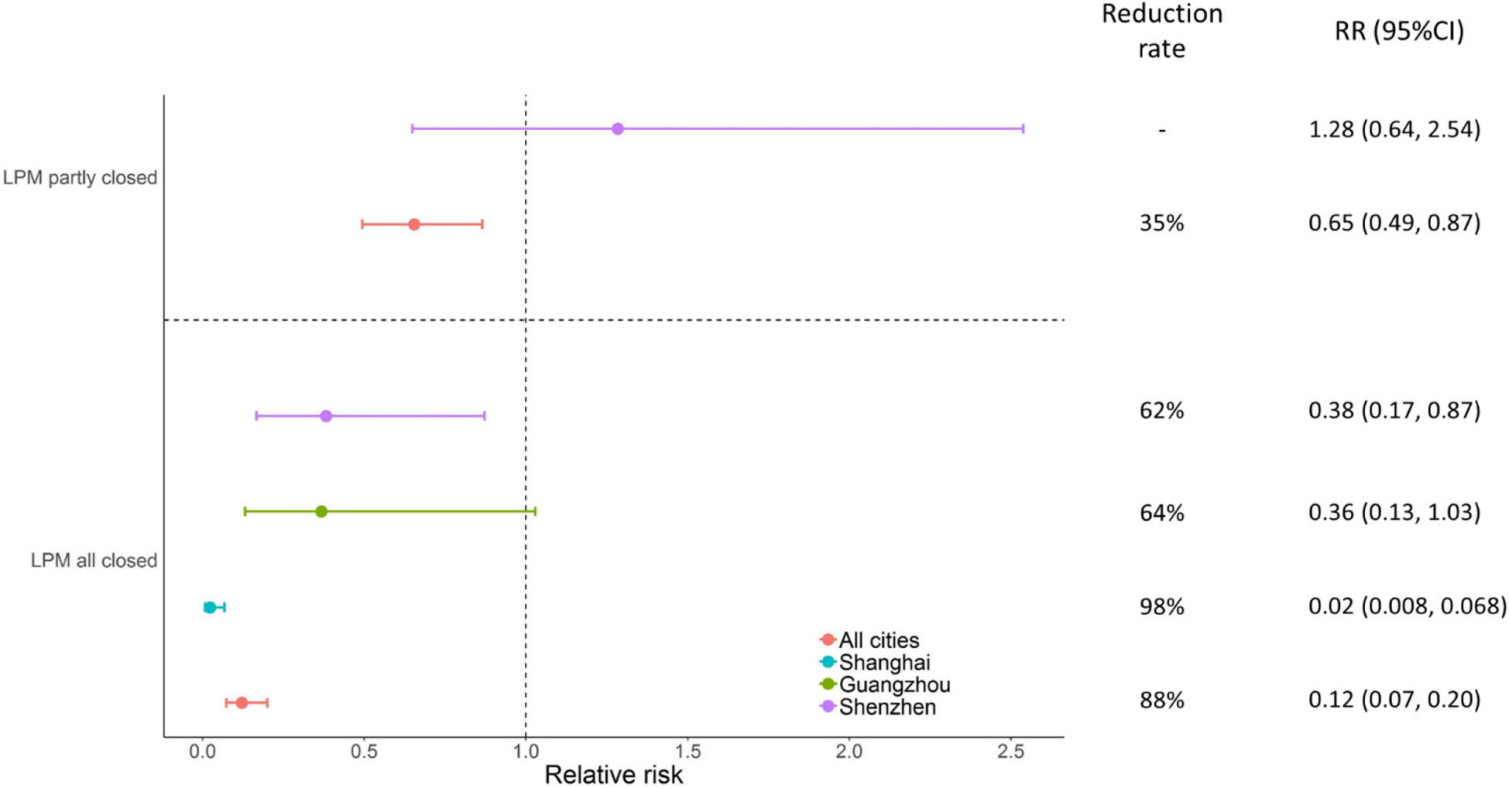
The relative risk (RR) plot for live poultry market (LPM) closure measure implemented in five cities with to reduce H7N9 cases. Each horizontal line represents a 95% confidence interval (CI). Risk reduction rate show in right

The relative risk (RR) of LPMs closure could be calculated from the period length of before, during and after intervention for each city (Fig. 4). Long-term LPMs closure in Shanghai induced a significant risk reduction of H7N9 cases infection (RR = 0.02, 95%CI = 0.008–0.068). Shenzhen adopted the partial LPMs closure was ineffective compared with all closure in the whole city (RR (95%CI) = 1.28 (0.64–2.54) vs. 0.38 (0.17–0.87)). For Guangzhou, however, implemented LPMs closure with a short time (2 weeks) period in the whole city, no significance for this measure (RR = 0.36, 95%CI = 0.31–1.03). Overall, the LPMs closures for controlling H7N9 infection was significantly reduced 88% relative risk during the intervention period for five cities, with RR ranging from 0.07 and 0.20 (*P* < 0.001).

### Classification and regression tree (CART) model

**Fig. 5:**
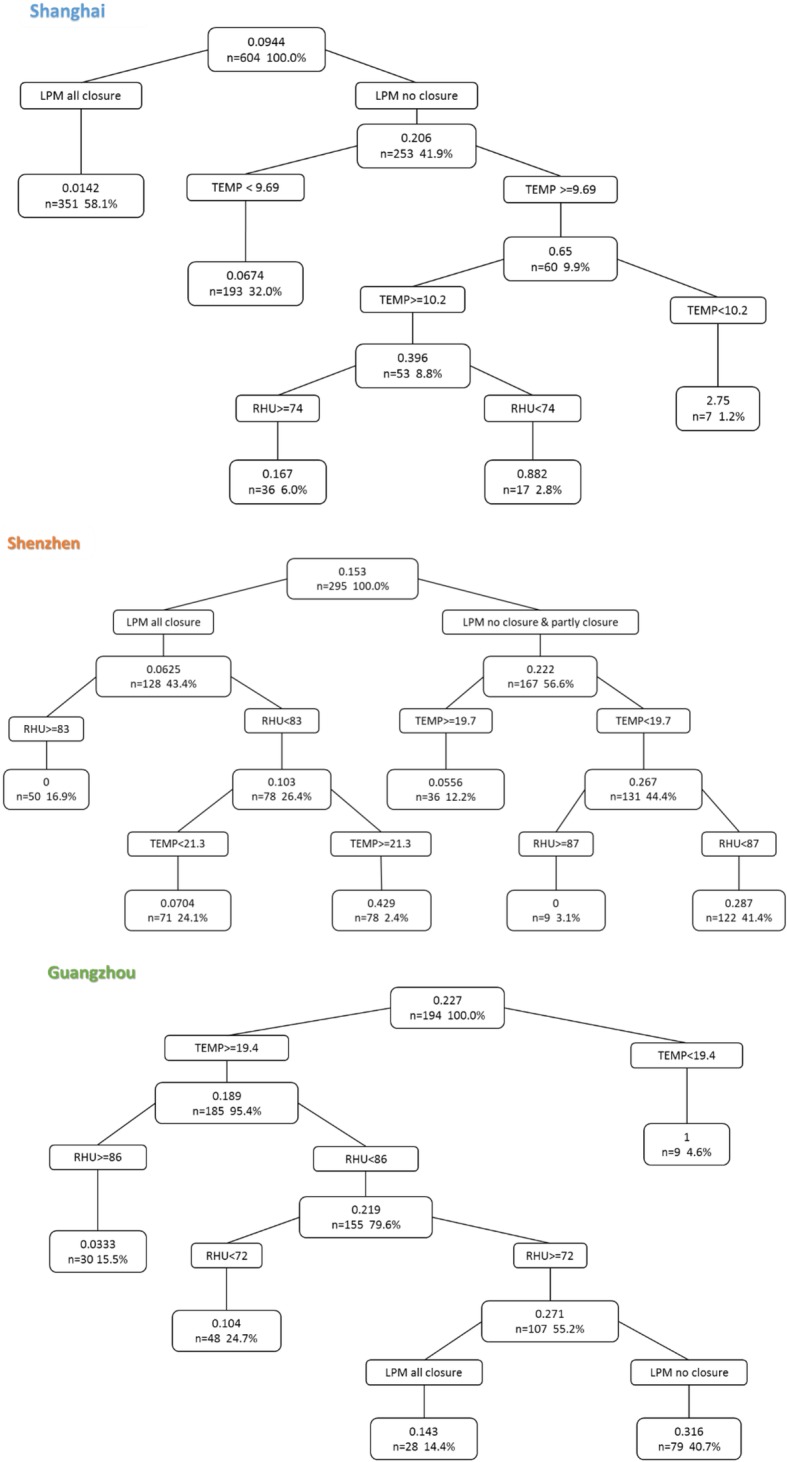
Classification and regression tree analysis of predictors of H7N9 cases due to measure of live poultry markets (LPMs) closure and meteorological factors. TEMP: temperature; RHU: relative humidity. Data are percentage of patients

We used CART analysis to detect the threshold for each factor with LPMs closure, temperature and relative humidity for H7N9 infection (Fig. 5). The tree of Shanghai showed the most significant and dominant factor was LPMs closure, which indicated that the daily incidence was 0.0142 when LPMs all closure, whereas daily incidence was 2.75 if LPMs no closure and temperature < 10.2 °C, or daily incidence was 0.882 if temperature > 10.2 °C and relative humidity < 74%. In Shenzhen, there was an almost 0 of daily incidence if LPMs all closure and relative humidity> = 83%, while daily incidence was 0.287 if LPMs no closure or partial closure, and relative humidity < 87% and temperature < 19.7 °C. The tree of Guangzhou indicated that LPMs closure was the last predict factor for H7N9 case infection, LPMs no closure with relative humidity from 72 to 86% and temperature > 19.4 °C led to 0.316 of daily incidence of H7N9 infection.

## Discussion

This is the first study compared the effectiveness of LPMs closure in cities that implemented the various scale and duration measure from first to fifth epidemics waves, for preventing human exposure to avian influenza H7N9 virus. Our study suggests that long-termed and fully closed such as Shanghai and Shenzhen (last two waves) have a most significant effect, while short-term closure has a limited effect such as Guangzhou, but partly closed even if long-termed closure was not significant such as Hangzhou and Suzhou. The results of classification tree analysis suggest that LPMs closure rather than environmental factors had the predominant role in reducing incidence for Shanghai and Shenzhen, which substantially controlled the H7N9 virus exposure to human.

How does the scope of LPMs closure affect? Yu and colleagues [8] demonstrated that closure of LPMs in four cities reduced the risk of human infections by 97–99% in June of 2013. It has indicated that LPMs closures were effective in controlling the human risk of H7N9 infection at that early time of H7N9 outbreak. But their study suggested that LPMs closure should be sustained in at-risk areas and implemented in any urban areas [8]. Our study’s results of Hangzhou and Suzhou city indicated if only urban area implemented the LPMs closure would not be effective to reduce the risk for human H7N9 infected, may due to the people who have the habit of eating live poultry are still willing to the suburb or rural area to purchase the live poultry. In one investigated study [23] convincingly demonstrated this fact that all respondents were asked about, without consideration of other factors, which type of the chicken they preferred most among the four types of chicken (i.e. live, freshly slaughtered, chilled or frozen). A significant portion of 47% of respondents indicated that they preferred live chicken above other forms of chicken, while 32% expressed “indifferent”. Live poultry is a necessity for many residents and related workers, that may explain why partial closure does not cause have an ideal controlling consequent. The separate area to conducted LPMs closure in a city cannot really block the exposure of live poultry.

How does the duration of LPMs closure affect? Many published studies agreed that the government would play a crucial role to implement the policy of LPMs closure timely once a confirmed case has occurred in this city [24]. Some even suggested that LPMs should be closed immediately in areas where H7N9 virus is detected in LPMs [25]. From a public health standpoint, LPMs should remain closed in all affected areas until the current influenza A (H7N9) epizootic and epidemic are declared definitively over by national authorities [8]. Moreover, one study suggested that A (H7N9) virus activity peaked around March in both retail and wholesale LPMs, while A (H5N1) virus peaked around in December to January in China [6]. Based on the evidence of our results, control measures (e.g. LPMs all closure) for avoiding exposure from live poultry to human and surveillance in LPMs should be maintained over spring.

We adjusted the influence of meteorological factors in generalized linear regression, and added temperature and relative humidity as predictors in the regression tree because meteorological factors are also one of the factors that affect the incidence of H7N9. Previous studies have indicated that odds ratio of precipitation (49.19–115.60 mm), sunshine (22–9.25 h), temperature (< 9.33 °C) [26] and wind speed (2.1–3.0 m/s or 6.3–7.1 m/s) [4] was statistically significant for H7N9 incidence. Both daily minimum and daily maximum temperature contributed significantly to human infection with the influenza A H7N9 virus [27], and the temperature range is similar to our study. Other study shown the risky windows of H7N9 infection were different in the northern and southern areas [28]. This point is consistent with our study. The temperature and humidity ranges that increase the risk of H7N9 vary from city to city.

However, Whether LPMs can be reopened to satisfy the Chinese tradition of buying live poultry when there is no H7N9 outbreak, this issue is still worth discussing. Related study demonstrated the poultry industry loosed amounted to ¥7.75 billion (USD$1.24 billion) in 10 affected provinces and ¥3.68 billion (USD$0.59 billion) in eight non-affected adjacent provinces in only 3 months from March to May, 2013 [16], included cost of poultry slaughter, cost of closing LPMs, cost of live poultry sales and cost of market-stall leases. After the H7N9 outbreak in whole of China, reduced to 6% of the household had experience of purchasing poultry [29]. Meanwhile, the proportion of direct medical losses and DALYs losses in the estimate of H7N9 burden was small, but the medical costs per case were extremely high (mean cost for each patient was ¥10,117 (USD$1619) for mild patients, and ¥205,976 (USD$32956) for severe cases with death).

Besides the serious agriculture losses [30], the effectiveness of LPMs needs further evaluation. One field study demonstrated that during the closure, H7N9 viral RNA detection and isolation rates in retail markets decreased by 79 and 92%, respectively. However, viable H7N9 virus could be cultured from wastewater samples collected up to 2 days after the market closure began [31]. Overall, LPMs reopen and temporary interventions cannot completely halt A (H7N9) virus persistence and dissemination. In order to balance the interests of agriculture economics [32] and public health, government encourages residence purchase chilled chicken instead of live poultry. Chilled chickens are slaughtered and packaged chicken stored at between 0 °C and 4 °C after going through a chilling process and has a shelf-life of 5–7 days, which can highly reduce the risk of virus transmission from live poultry to human [23]. Since the outbreaks of H7N9, many cities that reported the cases have launched a pilot program (named “Centralized Slaughtering, Cold Chain Delivery, Chilled Poultry Supply”) in designated areas, such as 2013 in Shanghai, 2014 in Guangzhou and Shenzhen [11]. Furthermore, the long-lasting circulation of the H7N9 virus in market poultry has often preceded the emergence of H7N9 outbreaks [14]. Based on the above considerations, on 1st June 2016, Shenzhen was the first city in China implemented the LPMs all closure in the whole city and sold chilled poultry instead of live poultry in urban and suburb areas. This policy of live poultry ban in the whole city can prevent any type of avian influenza not only H7N9. It is an optimal measure with long-term effectiveness to block the avian infected virus transmit to human, our results proved that as well.

Furthermore, in September 2017, an H5/H7 bivalent inactivated vaccine for chickens was introduced, and the H7N9 virus isolation rate in poultry dropped by 93.3% after vaccination. Only three H7N9 human cases were reported between October 1, 2017 and September 30, 2018 (the sixth epidemics period), indicating that vaccination of poultry successfully eliminated human infection with H7N9 virus [33]. Recently, however, there are thousands of pneumonia patients in Wuhan, a city in central China. The symptoms are very similar to SARS in 2013. A Novel coronavirus was isolated from the patients which denoted 2019-nCoV by the WHO. At first, the common point of the sick patients was that they had been to the Huanan Seafood Wholesale Market in Wuhan, which also sold live wild animals. This market was later identified as the center of a respiratory illness outbreak by health authorities [34]. This incident further reminds us that the sale of live wild animals or poultry and animals poses a great threat to public health.

Our study has some limitations. As an ecologic study, our findings might be affected by other potential confounding (e.g., human movement etc.). If we were able to collect the samples from the environment around the live poultry markets to detect the positive rate of virus of LPMs in every city, it can be a piece of strong evidence to support our results. Despite poultry immunity has been controlled, it does not rule out that the evolutionary genes transmit into human beings or emerge other types of avian influenza. It is necessary to adopt long-term, large-scale LPMs closure or even permanent ban on the sale of live poultry and wild animal in high-risk area and densely populated cities.

## Conclusion

Our findings suggest that LPMs all closure in whole city can be a highly effective measure comparing with partial closure (i.e. only urban area closure, suburb and country remain open) for preventing human exposure to avian influenza H7N9 virus. To extend the closure duration and consider permanently closing the LPMs will help improve the control effect. The effect of LPMs closure is greater than the impact of meteorology on the H7N9 human cases infection.

## Data Availability

The data that support the findings of this study are available from the Chinese National Influenza Centre and the Centre for Health Protection of Hong Kong but restrictions apply to the availability of these data, which were used under license for the current study, and so are not publicly available. Data are however available from the authors upon reasonable request and with permission of Chinese National Influenza Centre and the Centre for Health Protection of Hong Kong.

## Abbreviations

LPMs: Live poultry markets
RRR: Relative risk reduction
CARTs: Classification and regression trees
TEMP: Daily mean temperature
RHU: Relative humidity
RR: Relative risks
USD: USA dollar
